# Parallel RNA extraction using magnetic beads and a droplet array

**DOI:** 10.1039/c4lc01111b

**Published:** 2014-12-18

**Authors:** Xu Shi, Chun-Hong Chen, Weimin Gao, Shih-hui Chao, Deirdre R. Meldrum

**Affiliations:** a Center for Biosignatures Discovery Automation , The Biodesign Institute , Arizona State University Tempe , Arizona , USA . Email: joe.chao@asu.edu; b Department of Electrical Engineering , National Cheng Kung University Tainan , Taiwan

## Abstract

Nucleic acid extraction is a necessary step for most genomic/transcriptomic analyses, but it often requires complicated mechanisms to be integrated into a lab-on-a-chip device. Here, we present a simple, effective configuration for rapidly obtaining purified RNA from low concentration cell medium. This Total RNA Extraction Droplet Array (TREDA) utilizes an array of surface-adhering droplets to facilitate the transportation of magnetic purification beads seamlessly through individual buffer solutions without solid structures. The fabrication of TREDA chips is rapid and does not require a microfabrication facility or expertise. The process takes less than 5 minutes. When purifying mRNA from bulk marine diatom samples, its repeatability and extraction efficiency are comparable to conventional tube-based operations. We demonstrate that TREDA can extract the total mRNA of about 10 marine diatom cells, indicating that the sensitivity of TREDA approaches single-digit cell numbers.

## Abstract 2

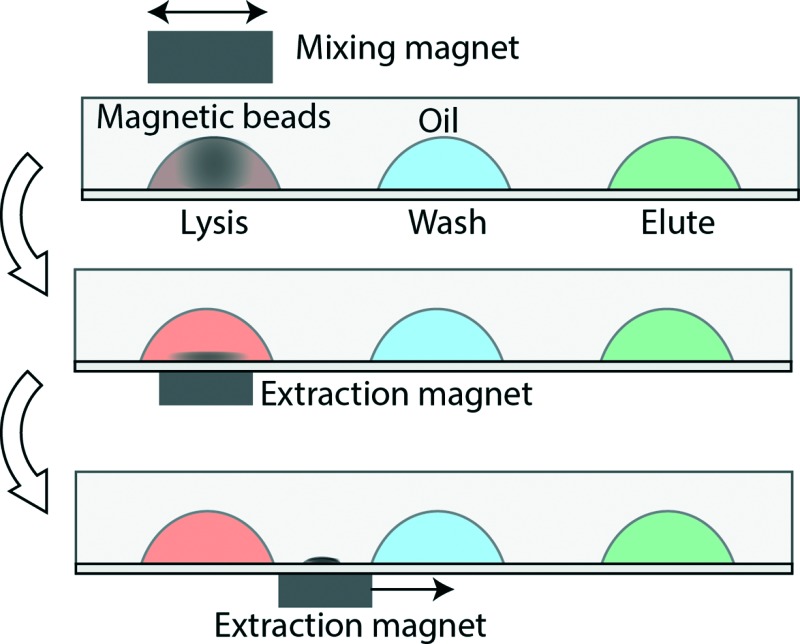
Nucleic acid extraction is a necessary step for most genomic/transcriptomic analyses, but it often requires complicated mechanisms to be integrated into a lab-on-a-chip device.

## Introduction

Currently, typical bench-top purification methods for nucleic acids are performed in either tubes or wells in microplates using solid phase extraction (SPE). These tubes or wells contain purification columns with large surface areas whose affinity to the targeted nucleic acids can be manipulated. These conventional bench-top purification processes are usually labor-intensive. Robotic workstations that use 96-well microplates exist; however, they are typically not accessible to general biologists and utilize liquid volumes larger than 10 μL. In addition, the cost of reagents and operation of large-scale robotic purification is high, and sensitivity for detecting low abundant targets is limited because of low template concentration.

Generally, purified nucleic acids are a prerequisite for performing downstream analysis, such as quantitative PCR (qPCR), reverse transcriptase quantitative PCR (RT-qPCR) and DNA/RNA sequencing. Some platforms such as Fluidigm's C1 (Fluidigm, S. San Francisco, CA), a commercial system which provides parallel pre-qPCR preparation for single mammalian cells, adopt all-in-one qPCR/RT-qPCR processes to eliminate DNA/RNA purification. These processes typically use lysis master mixtures that inhibit DNase or RNase activity to prevent degradation of the nucleic acids, and the components in the mixtures do not inhibit PCR/RT-PCR. However, some non-mammalian cells such as diatoms and most gram-positive bacteria have stronger cellular membranes, thus the necessary lysis buffer is no longer compatible with downstream PCR.^1^ For general applications, a separate step for nucleic acid extraction and purification is still required.

Initial efforts to perform on-chip nucleic acid extraction and purification incorporated purification columns with packed beads,^2–4^ porous polymers,^5–10^ or sol–gel matrices.^11^ These devices have complicated channel networks and on-chip valves to direct various buffers to the columns during the purification process. Therefore, the devices in the literature are limited in the number of purification reactions performed on one chip. Zhang *et al.* ^12^ developed a microwell system to perform RNA purification based on liquid phase extraction. This system does not need on-chip columns so it can perform multiple purification reactions simultaneously, but sophisticated liquid/air control valves are needed.

Purifying DNA/RNA using magnetic beads to retain and then elute nucleic acids can be compatible with microfluidic platforms and operate in parallel. These beads are commercially available with a variety of affinity options. Beads bound with nucleic acids can be easily immobilized by a nearby magnet. Therefore, the magnetic bead-based method eliminates the need for fabricating structures for on-chip columns required for each purification reaction and reduces the complexity of fabrication.^13^ Application of an external magnetic field causes the magnetic beads to form a bead aggregation in a section of a microchannel.^2,14^ The remaining purification processes are very similar to those of on-chip columns. For example, Quake's group uses on-chip pneumatic fluidic control valves to deliver lysis buffer, wash buffer, and elution buffer, to the retained magnetic beads and to purify mRNA from single mammalian cells.^15^ Lee's group also makes similar devices with integrated electromagnets to manipulate magnetic beads.^16,17^ RNA purification devices using magnetophoresis with continuous flow are also demonstrated.^18–20^ In these cases, RNA binding magnetic beads in continuous flow pass by an adjacent magnet, so the trajectories of the beads are deflected into a branch channel and separated from the main stream. However, the use of continuous flow is not compatible with applications that favor liquid segmentation (such as single cell RNA analysis).

Combining magnetic beads and microdroplets is gaining researchers' interest. Magnetic beads can penetrate through the oil/droplet interface through the use of an external magnetic field, so this class of devices does not need extensive microchannel networks for liquid manipulation. Simple structures reduce the cost needed for the extraction process and increase the number of reactions on a chip. Lehmann *et al.* ^21^ use an array of planar electromagnets to move and merge magnetic bead-containing droplets on a Teflon-coated surface, and employ hydrophilic/hydrophobic patterning to facilitate droplet splitting. Because magnetic fields do not interfere with static electric fields, magnetic separation has been integrated with electrowetting on dielectric (EWOD) droplet manipulation.^22,23^ Among these EWOD devices, Ohashi *et al.* ^23^ demonstrated that magnetic beads can move between two stationary droplets which are held by two sets of circular electrodes. A drawback of these EWOD-magnetic hybrid devices is that the electrodes require special surface coatings and electrical circuitries to manipulate the motion of the droplets, making the fabrication process more complicated. An alternative droplet-based method to EWOD is to use specially designed structures that can hold droplets but let magnetic beads traverse through gaps in the structures. Gu *et al.* ^24^ use external magnets to drag magnetic beads through a series of stationary picoliter droplets which contain different solutions for DNA purification in capillary tubing. The authors successfully purified the DNA of single mammalian cells. Similar approaches to move magnetic beads through an array of droplets in a chip format have also been demonstrated, where the droplets were held by microfabricated obstructions (walls, chambers or columns) with small openings for beads to go through.^25,26^ mRNA extraction at the single-cell level has been achieved.^26^

Although the introduction of droplets significantly reduces the complexity of the device, the solid structures that hold droplets may also obstruct magnetic beads during manipulation. In this paper, we present a Total RNA Extraction Droplet Array (TREDA) that uses hydrophilic patterns to constrain aqueous solutions without physical obstruction. This approach not only reduces the fabrication processing required, but also removes the physical obstruction (walls or columns) that might also prohibit magnetic beads from moving, and easily performs RNA extraction in parallel for different samples. TREDA is similar to recently published devices that used magnetic beads to drag liquids through hydrophilic spots to perform complicated liquid manipulations,^27–29^ our device focuses on using hydrophilic spots to hold stationary droplets to facilitate extraction steps. These novel features enable rapid and inexpensive device fabrication, as well as provide efficient total RNA extraction. We demonstrate that this approach efficiently purifies RNA directly from a single digit number of marine diatom cells based on serial dilution. This improvement facilitates higher RNA recovery efficiency and leads to single cell level sensitivity.

## Materials and methods

### Experimental setup

**Fig. 1:**
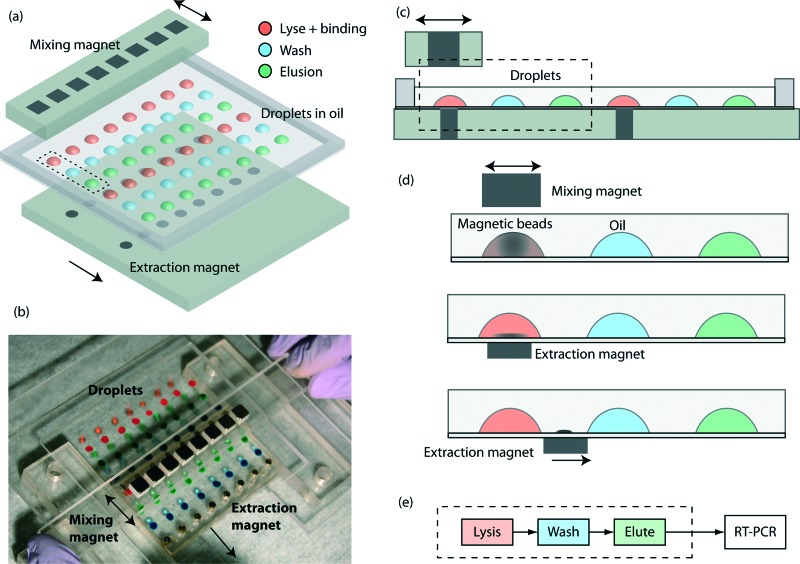
Overview of the TREDA system: (a) schematic of the device. An array of extraction magnets drags magnetic beads through droplets of lysis/binding buffer, wash buffer, and elution buffer. The current TREDA purifies 16 samples in parallel. The dotted rectangle encircles one “set” of purification droplets. (b) Picture of a TREDA chip on a manual processing station. Droplets are color-stained for illustration. A user is sliding an array of mixing magnets above the droplets. (c) Cross sectional view of TREDA when the chip is sitting on the extraction magnet plate. (d) Operational process: (1) add sample to droplets with preloaded lysis/binding buffer and magnetic beads. Slide the mixing magnet above the droplets to resuspend the magnetic beads. (2) Move the extraction magnets to right beneath the droplet to attract beads to the bottom. (3) Drag beads by moving the extraction magnets to the next droplet and then redo the process from d1 to d3 for all droplets. (e) The process workflow in the paper. The current TREDA encompasses the steps enclosed in the dashed box.

The key components are a TREDA chip, a movable Plexiglas plate embedded with two linear arrays of sixteen “extraction magnets” (D22-N52, 1/8" diameter × 1/8" thick, K&J Magnetics, Pipersville, PA) below the TREDA, and another movable Plexiglas bar with one linear array of eight “mixing magnets” (B444-N52, 1/4" cube, K&J Magnetics) above the TREDA (Fig. 1a). The TREDA chip holds 16 sets of surface adhering droplets to perform 16 simultaneous extractions, where each set comprises three droplets: one droplet for a mixture of lysis and binding buffer, one droplet for wash buffer, and one droplet for elution buffer. All droplets are submerged under an open pool of mineral oil confined by a PDMS frame. The formation of droplets is compatible with that of a standard 96-well microplate, so all droplets can be easily pipetted onto the TREDA chip using multichannel pipettes or robotic pipetting systems. The density of future TREDA chips can be increased with a highly packed array of small magnets. All droplets are confined in an array of hydrophilic spots on the glass surface.

The structure of the purpose-made device in the experiment is shown in Fig. 1b, where droplets were dyed with food coloring for visualization. The manipulation of the magnets is manually operated, but can be automated in the future. Fig. 1c shows the side view of two separated sets and their relative positions with the mixing magnets and the extraction magnets. Fig. 1d demonstrates the sequence of magnetic bead manipulation. First, with no extraction magnets, cells are introduced into the lysis/binding droplets (illustrated in red) and the mixing magnets oscillated above the TREDA array. With these steps, the beads in the droplets are suspended, oscillated and efficiently mixed with the cell lysates (Fig. 1d). After one minute, the mixing magnet bar is removed, and then the extraction magnets moved to the bottom of the chip to attract the beads in the droplets toward the bottom (Fig. 1d). The extraction magnets move the beads toward the washing droplet (illustrated in blue) while the lysis/binding droplet remains stationary because it is held within the hydrophilic area. Therefore, only the beads are transferred to the washing droplet (Fig. 1d). When beads enter the blue droplet, the same process is utilized for mixing and moving the beads. Lastly, the beads are moved away from the elution droplet (illustrated in green) and the purified RNA can be obtained. In the TREDA approach, lysis, wash and elution steps are completed on the chip. RT-qPCR is accomplished with a conventional in-tube reaction on the bench^30,31^ to quantify the RNA extracted/purified by the TREDA chip (Fig. 1e).

### Magnetic-bead extraction

Charge switchable magnetic beads (ChargeSwitch® Total RNA Cell Kits, Life Technologies, Carlsbad, CA) were used to extract RNA in this study. The beads carry positive charges when the surrounding pH is lower than 6.0, and negative charges when the pH is higher than 8.0. Therefore, naturally charged RNA can be bound or released from the surface of the beads based on the surrounding pH. A three-step purification method was used in this study. First, the cell medium, lysis buffer, binding buffer and beads were mixed together for cell lysis and RNA binding. The cells were lysed, and then the total RNA was bound onto the surface of the beads since the binding buffer has a low pH value. Second, the washing buffer was mixed to wash out the unwanted substances on the beads. Third, the low-salt, high pH elution buffer was mixed with the RNA-bound beads to release total RNA from the surface of the beads. After elution, the beads were removed, leaving purified total RNA in the elution buffer.

### Chip fabrication

The procedure of producing a hydrophilic array on the TREDA chip is derived from our previous work.^32^ The fabrication process is to make a hydrophilic pattern on silanized glass coverslips to confine the aqueous droplets. In this study, such patterns were generated by Microscale Plasma Activated Templating (μPLAT), a technique that employs a stencil to expose air plasma only to designed areas to increase the hydrophilicity of the surface.^33^ The TREDA chip was made on a no. 2, 76 mm × 89 mm, cover glass (260450, Ted Pella, Redding, CA). The hydrophilic pattern was determined using a 1 mm-thick PDMS stencil, which had an array of 8 × 6 1/8 inch-diameter holes, with a 9 mm pitch between centers to be compatible with standard 96-well microplates. The stencil was adhered on the coverslip and placed in a plasma cleaner (PDC-32G, Harrick Plasma, Ithaca, NY) for plasma exposure with 6.8 W RF-power for one minute. The areas exposed to the plasma became hydrophilic while the unexposed areas remained hydrophobic. Then the stencil was removed, leaving an array of hydrophilic patterns on a more hydrophobic background. The produced coverslip can hold 48 droplets. To confine oil during operation, a PDMS frame (76 mm × 89 mm with an 64 mm × 77 mm opening, 2 mm in thickness) was plasma bound on the edges of the chip to surround the array.

### Cell preparation

Marine diatom *Thalassiosira pseudonana* (CCMP1335) cells were grown in f/2 medium^34,35^ with constant light exposure. Artificial seawater was used for medium preparation based on the formula of Kester *et al.* ^36^ Cells were harvested and directly counted by using a Petroff Hausser counting chamber (Hausser Scientific, Horsham, PA). A serial dilution with 1× PBS was performed to generate around 10 000 cells μL^–1^, 1000 cells μL^–1^, 100 cells μL^–1^, 10 cells μL^–1^ and 1 cell μL^–1^, respectively. 1 μL of each concentration level was used as the starting material for on-chip RNA extraction.

### RNA extraction procedure (conventional approach)

Bench-top RNA extraction was performed according to the protocol provided by the manufacturer. In short, we freshly prepared lysis mix, which contained 5 μL 0.5M DTT and 5 μL 20 mg mL^–1^ proteinase K every 500 μL lysis buffer (L16). A 1 μL cell culture was directly lysed with this lysis mix and incubated at 60 °C for 15 min. 100 μL of magnetic beads and 200 μL of binding buffer (B9) were added into the cell lysates and gently mixed by using a pipette. A magnetic rack was used to form a tight pellet of beads and then the supernatant was removed by gently pipetting. 750 μL of wash buffer (W13) was added to wash the beads and followed by another washing step using 750 μL of wash buffer (W14). Supernatant was removed by using a magnetic rack. Final elution was achieved by adding 5 μL elution buffer (E7).

### RNA extraction procedure (on TREDA chip)

Sixteen 4 μL droplets and thirty-two 5 μL droplets were pipetted onto the TREDA chip. There were sixteen sets of droplets. Each set contained three droplets. The first droplet in each group contained a 4 μL mixture of 1 μL lysis Mix, 1 μL binding buffer, 1 μL cell medium and 1 μL suspension of magnetic beads. The second and the third droplets contained 5 μL washing buffer and 5 μL elution buffer, respectively. After pipetting the droplets onto the TREDA chip, 2.5 mL mineral oil (M5904, Sigma-Aldrich, St. Louis, MO) was loaded to cover the droplets inside the PDMS frame. The oil was utilized to prevent the droplets from evaporation and contamination. The RNA extraction process followed the description in the experimental setup section. The liquid in the third droplet was pipetted to a conventional PCR tube for RT-qPCR to validate RNA extraction efficiency.

### Reverse-transcription and qPCR processes

SuperScript VILO cDNA Synthesis kit was used for synthesis of cDNA (Life Technologies, Carlsbad, CA,) from RNA. Total reaction volume was 10 μL, which contained 1 μL 10× SuperScript Enzyme Mix, 2 μL 5× VILO Reaction Mix, 4 μL of DEPC treated water (Life Technologies) and 3 μL of eluted RNA. The thermal program for cDNA synthesis was as follows: 25 °C for 10 min, 42 °C for 2 hour and 85 °C for 5 min. For qPCR, primers were designed by using Primer-BLAST (http://www.ncbi.nlm.nih.gov/tools/primer-blast/) and manufactured by Invitrogen (Carlsbad, CA, US) which amplified a 184 bp of 18S of *T. pseudonana*: forward primer (5′-TGCCAGTAGTCATACGCTCGTCTCA-3′) and reverse primer (5′-CCTTCCGCGAACAGTCGGGTATT′). Express SYBR GreenER qPCR Supermix Universal kit (Life Technologies) was used in qPCR. Total reaction volume was 10 μL, which contained 1 μL of each primer with the concentration of 4 μM, 5 μL of EXPRESS SYBR GreenER qPCR SuperMix Universal, 0.1 μL ROX Reference Dye, 0.9 μL DEPC treated water and 2 μL cDNA. qPCR was performed on a StepOne Real-Time PCR System (Life Technologies, Carlsbad, CA) instrument. The negative controls were DEPC-treated RNA-free water. The thermal cycling program for qPCR was as follows: 95 °C for 5 min, 40 cycles for 95 °C for 15 s, 60 °C for 60 s and 75 °C for 10 s (signal collection), followed by melt curve analysis according to the default program of the instrument. Three replicates for each RNA sample extracted by the TREDA chip were performed.

## Results and discussion

### On-chip mixing

**Fig. 2:**
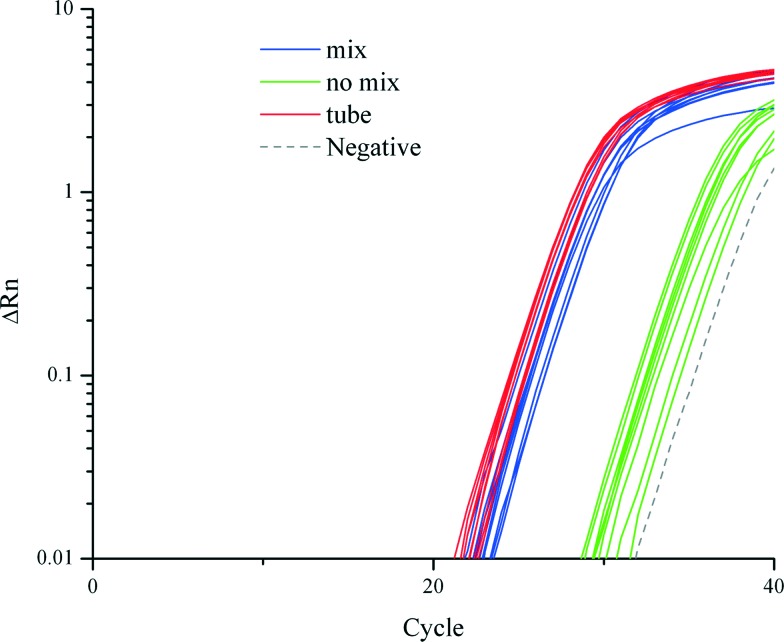
Comparison of RT-qPCR amplification curves from conventional tube-based method, and our TREDA chip with and without mixing.

We demonstrated that cell lysis, RNA extraction and purification on a TREDA chip with mixing were as efficient as conventional approaches which used vortexing or pipetting. Fig. 2 shows the RT-qPCR results on 18S RNA that compares three purification approaches: the conventional tube based method directly follow the vendor's protocol, TREDA with mixing, and TREDA without mixing. Conventional purification was performed in Eppendorf tubes following Life Technologies' protocol and using an original magnetic rack (CS15000). The sample was 1 μL cell medium containing around 100 *T. pseudonana* cells. The PCR primers target 18S RNA. The blue and green amplification curves represent the use of TREDA with and without the mixing process, respectively. In this experiment, we performed three purification experiments for each purification approach (*i.e.*, three groups of droplets for TREDA or three purification tubes for conventional) so the triplicate results indicate the technical variations of purification. Each purified droplet was further partitioned into three Eppendorf tubes for RT-qPCR. The triplicate RT-qPCR results of the same purification indicate the technical variations of PCR. Therefore, each approach has 9 curves and 1 curve for negative control. The average *C*_q_, quantification cycle represents the number of cycles needed to reach a set threshold fluorescence signal level. The average *C*_q_ value of the conventional approach was 26.5, and for our approach with the mixing process *C*_q_ was 27.5, and for our approach without the mixing process *C*_q_ was 34.6. Standard deviations of the conventional approach, our approaches with and without the mixing process were 0.39, 0.53 and 0.88, respectively. The results indicate the comparable quantity and quality between our approach and the conventional approach and the necessity of the mixing.

In RNA extraction without the mixing process, magnetic beads attached to the chip surface in all processes due to the extraction magnet. When utilizing the mixing process, the beads are attracted from the chip surface to the top of the droplet. The mixing increases the contact surface between the beads and liquid which improves the mixing efficiency. Therefore, the average *C*_q_ value of the mixing curves is much higher than that of the no mixing curves. The negative control shows amplification in the late stage due to random nonspecific amplifications after extended thermocycling. The melting curve of the negative control can be easily distinguished from the ones with specific amplifications.

### Efficiency, sensitivity, and working range of the method

**Fig. 3:**
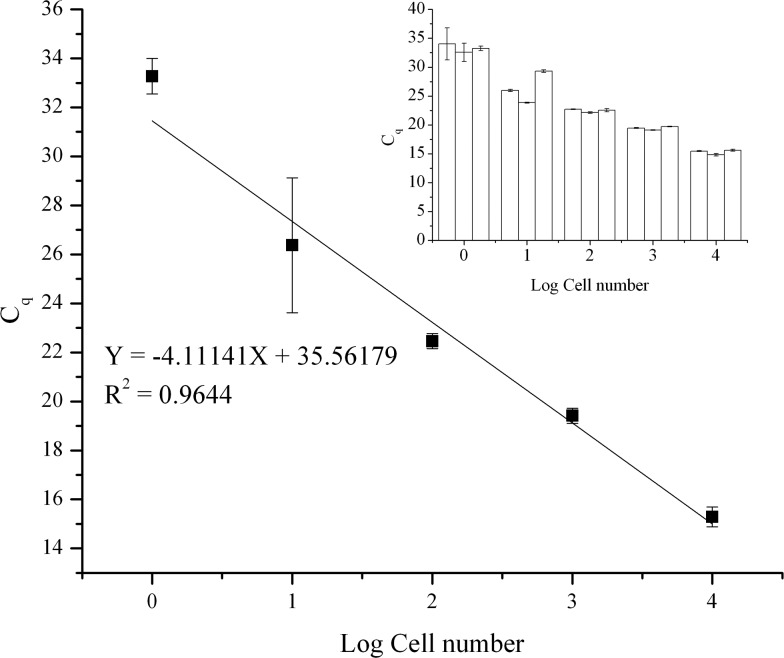
Purified qPCR standard curve and statistics of *C*_q_ values as functions of cell numbers.

The efficiency, sensitivity, and working range of the method using the TREDA chip with the mixing procedure have been evaluated. As shown in Fig. 3, five levels of cell numbers were tested, and the resultant qPCR data were used to generate the standard curve. Cell numbers of 10 000, 1000, 100, 10 cells and single cell per droplet (1e7, 1e6, 1e5, 1e4, 1e3 cells mL^–1^) were achieved by serial dilution from bulk cells. There were triplicates for each cell concentration level. We used these triplicate results to identify variations in the purification process. In addition, we partitioned each purified RNA into three tubes for RT-qPCR. We further used the triplicate RT-qPCR results to identify the variation of the PCR process. The small graph in the upper right corner represents the average *C*_q_ value and variation in each set. The average *C*_q_ values from low concentration to high concentration are 33.3, 26.4, 22.5, 19.4, 15.3, respectively. Negative controls were not detected. The standard deviations from a low number to a high number of cells are 0.73, 2.75, 0.30, 0.30 and 0.40, respectively. The result shows our approach has high sensitivity to obtain RNA from a single cell. In addition, the result shows our method has a wide working range since there is a high linear relationship between the average *C*_q_ value and the cell number. However, compared to higher cell concentrations, the standard deviation increases significantly at 10 cell and single cell levels, which have higher *C*_q_ values. Since PCR becomes more stochastic in the presence of fewer templates, the higher variation seen with higher *C*_q_ values is an inherent characteristic of the PCR process.^31^

## Conclusion

We presented a novel, magnetic bead-based RNA purification chip called TREDA with a series of surface tension-confined droplets. We demonstrated this device can efficiently purify RNA from raw diatom cells ranging from 10 000 cells to single cells. The RT-qPCR reactions performed on the purified RNA show consistent, sensitive data with low variation which confirmed the high quality of RNA purified by our TREDA chip. The constructional and operational simplicity of the chip makes it a robust tool for RNA purification. It will save significant time and minimize human errors due to reduced handling steps. This droplet-based purification format can be extended to measure quantitative gene expression if one of the droplets in the reaction contains RT-qPCR reagents. The design is an ideal format for coupling with the qPCR arrays that utilize surface-adhering droplets.^32^ Our team is developing a similar chip with a droplet array for performing RT-qPCR, which can be readily integrated with TREDA. TREDA can be integrated with other RT-qPCR platforms, *e.g.*, loading the extracted RNA into tubes then perform RT-qPCR on commercially available thermocyclers. Additionally, this device is able to provide high quality purified RNA for other downstream analysis, such as RNA-Seq, regardless of the initial cell concentration. This property will assure the reliability of RNA-Seq results, especially for single cell analysis.
